# Functional nucleic acid engineered double‐barreled nanopores for measuring sodium to potassium ratio at single‐cell level

**DOI:** 10.1002/EXP.20220025

**Published:** 2022-05-23

**Authors:** Xiao‐Mei Shi, Fang‐Qing Liu, Bing Wang, Si‐Yuan Yu, Yi‐Tong Xu, Wei‐Wei Zhao, Dechen Jiang, Hong‐Yuan Chen, Jing‐Juan Xu

**Affiliations:** ^1^ State Key Laboratory of Analytical Chemistry for Life Science School of Chemistry and Chemical Engineering Nanjing University Nanjing P. R. China

**Keywords:** θ‐nanopipette, ionic current rectification, nucleic acid, single cell, sodium to potassium ratio

## Abstract

The use of double‐barreled nanopipette (θ‐nanopipette) to electrically sample, manipulate, or detect biomaterials has recently seen strong growth in single‐cell studies, driven by the potential of the nanodevices and applications that they may enable. Considering the pivotal roles of Na/K ratio (R_Na/K_) at cellular level, herein we describe an engineered θ‐nanopipette for measuring single‐cell R_Na/K_. The two independently addressable nanopores, located within one nanotip, allow respective customization of functional nucleic acids but simultaneous deciphering of Na and K levels inside a single cell of a non‐Faradic manner. Two ionic current rectification signals, corresponding to the Na‐ and K‐specific smart DNA responses, could be easily used to derive the R_Na/K_. The applicability of this nanotool is validated by practical probing intracellular R_Na/K_ during the drug‐induced primary stage of apoptotic volume decrease. Especially, the R_Na/K_ has been shown by our nanotool to be different in cell lines with different metastatic potential. This work is expected to contribute to futuristic study of single‐cell R_Na/K_ in various physiological and pathological processes.

## Graphical abstract

Functional nucleic acid engineered double‐barreled nanopores were designed and applied for measuring Na/K ratio (R_Na/K_) at single‐cell level. The applicability of this nanotool is validated by practical probing intracellular R_Na/K_ during the drug‐induced primary stage of apoptotic volume decrease. Especially, the R_Na/K_ has been shown by our nanotool to be different in cell lines with different metastatic potential.

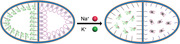

## 1 INTRODUCTION

Sodium (Na) and potassium (K) function in tandem throughout the human body and the Na/K ratio (R_Na/K_) is crucial for our health.^[^ ^1^ ^]^ At the macroscopic level, poor R_Na/K_ has been linked to severe pathological consequences such as hypertension, obesity, stroke, cardiovascular and urinary stone disease, and even all‐cause mortality.^[^ ^2^ ^]^ At the microscopic level, regulated by the Na‐K pump in the outer cell membrane, every cell in our body necessitates a proper intracellular R_Na/K_, that is, ∼15 ± 5 mM Na versus ∼125 ± 25 mM K, to support the normal cellular physiology.^[^ ^3^ ^]^ Disturbed cellular R_Na/K_ has been correlated to generation of electrical transmembrane potentials and probably involved in control of mitogenesis and cell division.^[^ ^4^ ^]^

Due to the cellular heterogeneity, single‐cell studies have drawn enormous attention with the aim to promote in‐depth understanding of fundamental physiology and precise pathological diagnoses and therapeutic trials.^[^ ^5^ ^]^ Fluorescent and electrochemical techniques have been increasingly exploited to measure important endogenous species inside single living cells. However, this is not the case for R_Na/K_. Optically, the bottleneck is due to the difficulty of developing superb fluorescent probes that are respectively selective to Na and K without spectral overlap.^[^ ^6^ ^]^ Electrochemically, the ion‐selective microelectrodes are plagued by their sizes for subcellular applications,^[^ ^7^ ^]^ while the potential‐resolved nanoelectrodes^[^ ^8^ ^]^ are limited by the impotence of potential‐directed differentiation of the two ions. So far, the measurement of single‐cell R_Na/K_ is still not tackled.

Ionic current rectification (ICR) represents asymmetric transportation property of ions within nanoscale charged channels, which induces the ionic rectification as indicated by the potential‐dependent asymmetry of the *I*–*V* curves.^[^ ^9^ ^]^ Recently, ICR‐nanopipette technique has been shown to be promising toward single‐cell studies,^[^ ^10^ ^]^ which combines the advantage of nanopipette as a transmembrane vector of minimal invasiveness and the sensitivity of ICR with a non‐Faradic operation. Despite its attractive capacities, due to its short development time, to date only limited studies have been conducted with the use of single‐barrel nanopipette addressing intracellular small biomolecules,^[10a^ ^–^ ^10f^ ^]^ single ions,^[10g^ ^–^ ^10i^ ^]^ and RNA.^[10j^ ^]^ On the other hand, double‐barreled nanopipette (θ‐nanopipette), consisting of two independent apertures that are situated at its nanotip and separated by a small septum, has recently been proven as a powerful tool for single‐molecule manipulation,^[^ ^11^ ^]^ single‐cell sampling^[^ ^12^ ^]^ and single‐nanoparticle detection.^[^ ^13^ ^]^ Inspired by the accessibility of two individual nanopores, we hypothesize that appropriate tailoring of the two nanopores with ion‐specific smart DNA could create novel ICR‐nanopipette tool toward single‐cell R_Na/K_ measurement.

**FIGURE 1:**
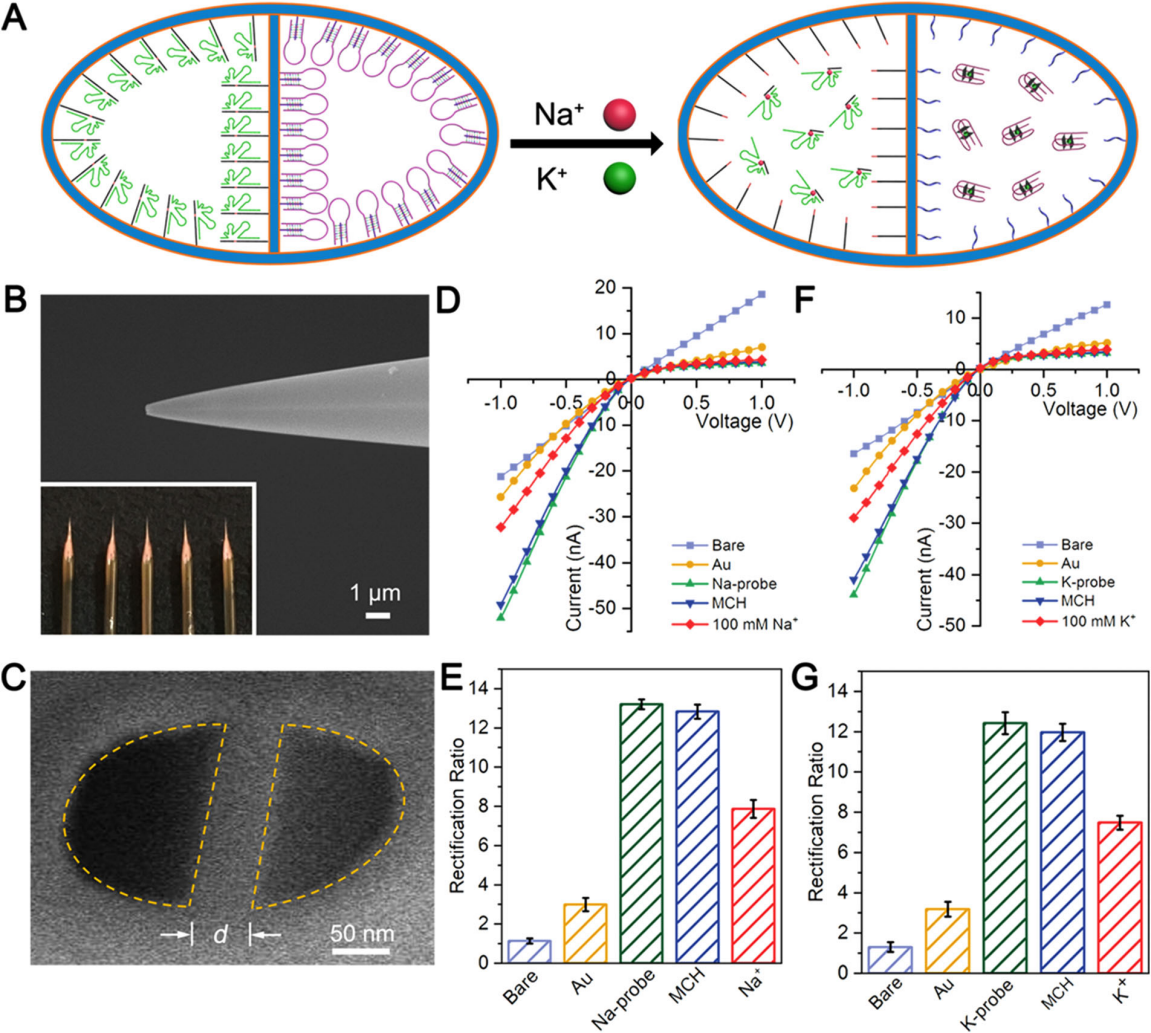
(A) Schematic illustration of the smart DNA engineered θ‐nanopipette for probing R_Na/K_. SEM images of (B) the side‐ and (C) top‐view of the θ‐nanopipette. Inset below: digital photographs of the θ‐nanopipettes; Stepwise *I*–*V* curves and the corresponding ICR ratios in the development of (D,E) left Na‐nanopore and its responses to 100 mM Na^+^ and (F,G) right K‐nanopore and its responses to 100 mM K^+^. The supporting electrolyte contained 20 mM Tris‐HCl with LiCl of 40 mM (pH = 7.4). Error bars represent standard deviations from three independent tests

Here we report a DNA engineered ICR‐θ‐nanopipette to this end (see Supporting Information for experimental details). The θ‐nanopores were created by laser‐pulling of θ‐type capillaries and the interior walls were then sputtered with Au coating (Figure S1). As illustrated in Figure 1A, the left nanopore was functionalized with NaA43SE DNAzyme with Na^+^‐selectivity (denoted as Na‐nanopore), while the right nanopore was functionalized with triple‐helix molecular switch with K^+^‐selectivity (denoted as K‐nanopore). In responding to Na^+^ and K^+^, partial DNA sequences would respectively depart from the interior walls of the Na‐nanopore and K‐nanopore. Because of the polyanion nature of DNA, the alternation of the charge property would sensitively induce the ICR change for R_Na/K_ derivation. Upon drug stimulations, intracellular R_Na/K_ studies during the primary stage of apoptotic volume decrease (PSAVD) confirmed the practical applicability of this nanotool with minimal invasiveness and cell‐context preservation. Especially, we found the different R_Na/K_ in cells with different metastatic potential.

## 2 RESULTS AND DISCUSSION

### 2.1 Fabrication of the single‐cell R_Na/K_ nanotool

Figure 1B and inset show the side‐view scanning electron microscope (SEM) and optical images of the as‐pulled θ‐nanopipette, while Figure 1C shows the top‐view SEM image of the nanotip that consisted of two coplanar semi‐elliptical nanopores. The left and right pores were of 125 ± 11 nm and 120 ± 7 nm (mean ± SD, *n* = 10), respectively, and separated by a septum of 50 ± 5 nm (mean ± SD, *n* = 10). Gel electrophoresis confirmed Na^+^‐triggered responsibility of the DNAzyme^[^ ^14^ ^]^ and the K^+^‐triggered responsibility of the molecular switch (Figure S2),^[^ ^15^ ^]^ which were then respectively employed to establish the Na‐nanopore and K‐nanopore via the Au‐S bond, followed by the blocking of 6‐hydroxyl‐1‐hexanethiol (MCH). The stepwise development procedure and the Na^+^/K^+^ responses were monitored by *I*–*V* curves. As shown by the Na‐nanopore in Figure 1D, compared with the pristine nanopore (light blue curve), the Au deposition caused an increased ICR signal because of the strongly absorption of the chloride ion^[^ ^16^ ^]^ in LiCl electrolyte (orange curve). After the DNAzyme modification, the distinctly increased ICR ratio could be ascribed to the negatively charged DNA (lime curve),^[^ ^17^ ^]^ and the subsequent MCH blocking led to a slight signal variation (blue curve).^[^ ^18^ ^]^ Upon treatment by 100 mM Na^+^, the signal exhibited an obvious reduction because of the Na^+^‐triggered DNA release from the interior wall (red curve). Figure 1E shows the corresponding ICR ratios calculated as |*I*_−1.0 V_:*I*_+1.0 V_|. As shown by the K‐nanopore in Figure 1F,G, the ICR signals exhibited similar evolution and the K^+^‐induced ICR reduction was due to the release of K^+^ binding aptamer. The open‐circuit potential measurements were then performed to confirm the successful functionalization of the DNA sequences within the Au‐deposited θ‐nanopipette (Figure S3). Incidentally, the Au‐deposited θ‐nanopipette cannot cause any ICR change without Na‐probe and K‐probe modification, indicating only the specific interaction between the probes and target ions can cause the signal change (Figure S4).

**FIGURE 2:**
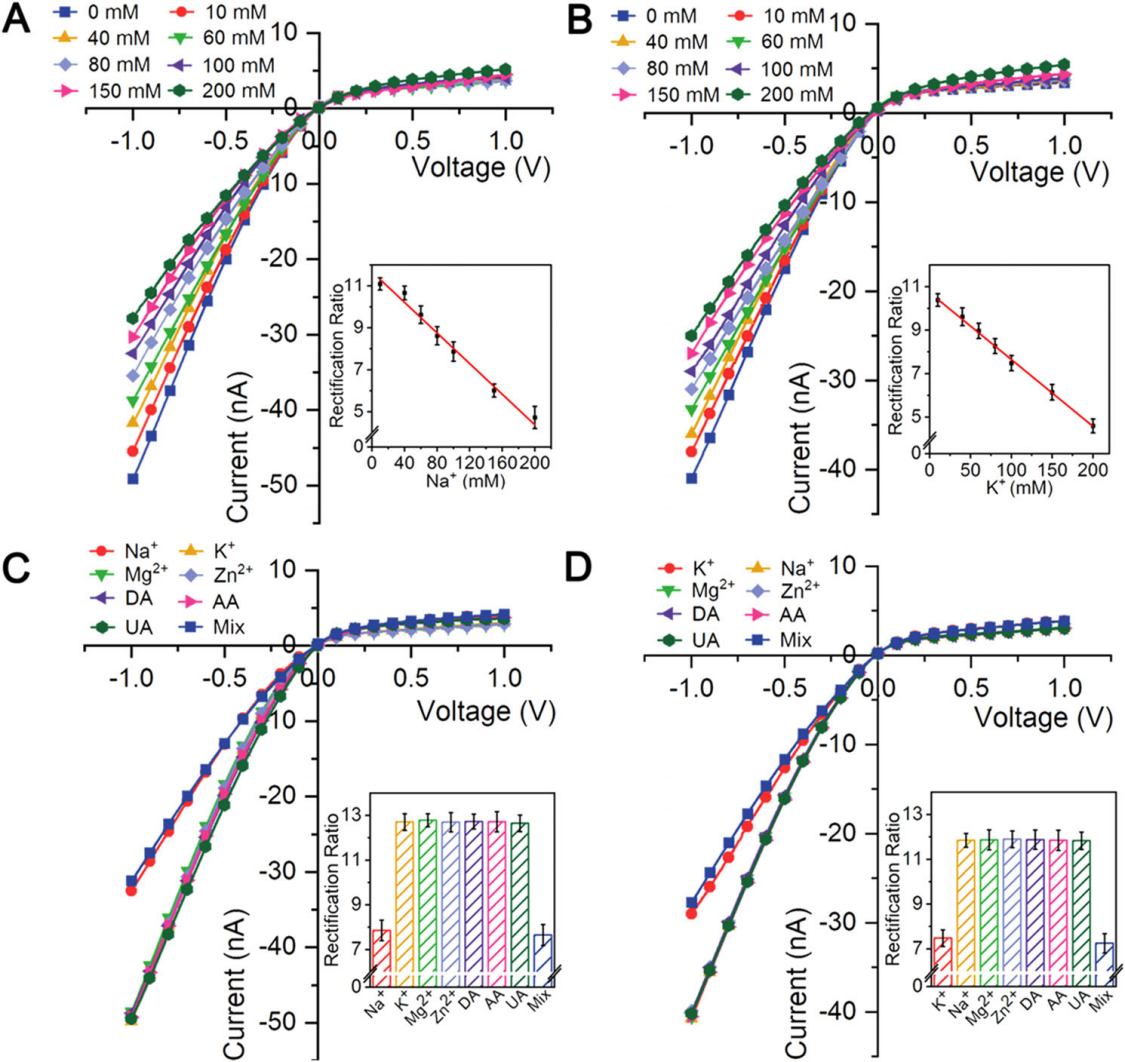
The *I*–*V* curves of R_Na/K_ nanotool toward different (A) Na^+^ or (B) K^+^ concentrations. Selectivity tests of (C) Na‐nanopore and (D) K‐nanopore toward various interference species and the mixed sample. Insets show the corresponding ICR ratios

The responses of the nanotool toward different Na^+^ or K^+^ concentrations were then investigated. As expected, in the range of 0 to 200 mM, the ICR signals exhibited gradually decrease with the increased Na^+^ (Figure 2A and inset) or K^+^ (Figure 2B and inset) concentrations, and the detection limits were calculated to be 1.25 mM for Na‐nanopore and 2.01 mM for K‐nanopore (S/N = 3), respectively. The selectivity studies were conducted by studying the responses toward 100 mM Na^+^ or K^+^ and different interference species including 5 mM of Mg^2+^, 180 nM of Zn^2+^,^[^ ^19^ ^]^ 100 µM dopamine, ascorbic acid, and uric acid^[10a^ ^]^ as well as their mixture. As shown, the Na‐nanopore (Figure 2C and inset) and K‐nanopore (Figure 2D and inset) only respectively responded to the Na^+^ and K^+^, even in the mixture, indicating the good selectivity of the nanotool. Incidentally, the stability of the nanotool was also confirmed (Figure S5). The incubation time for Na^+^ and K^+^ reactions was optimized (Figure S6). Besides, all of the as‐prepared nanotools were screened before practical usage to guarantee the precision of studies (Figure S7). The results above indicated the potential of the as‐prepared nanotool for practical R_Na/K_ probing.

### 2.2 In vivo probing single‐cell R_Na/K_

**FIGURE 3:**
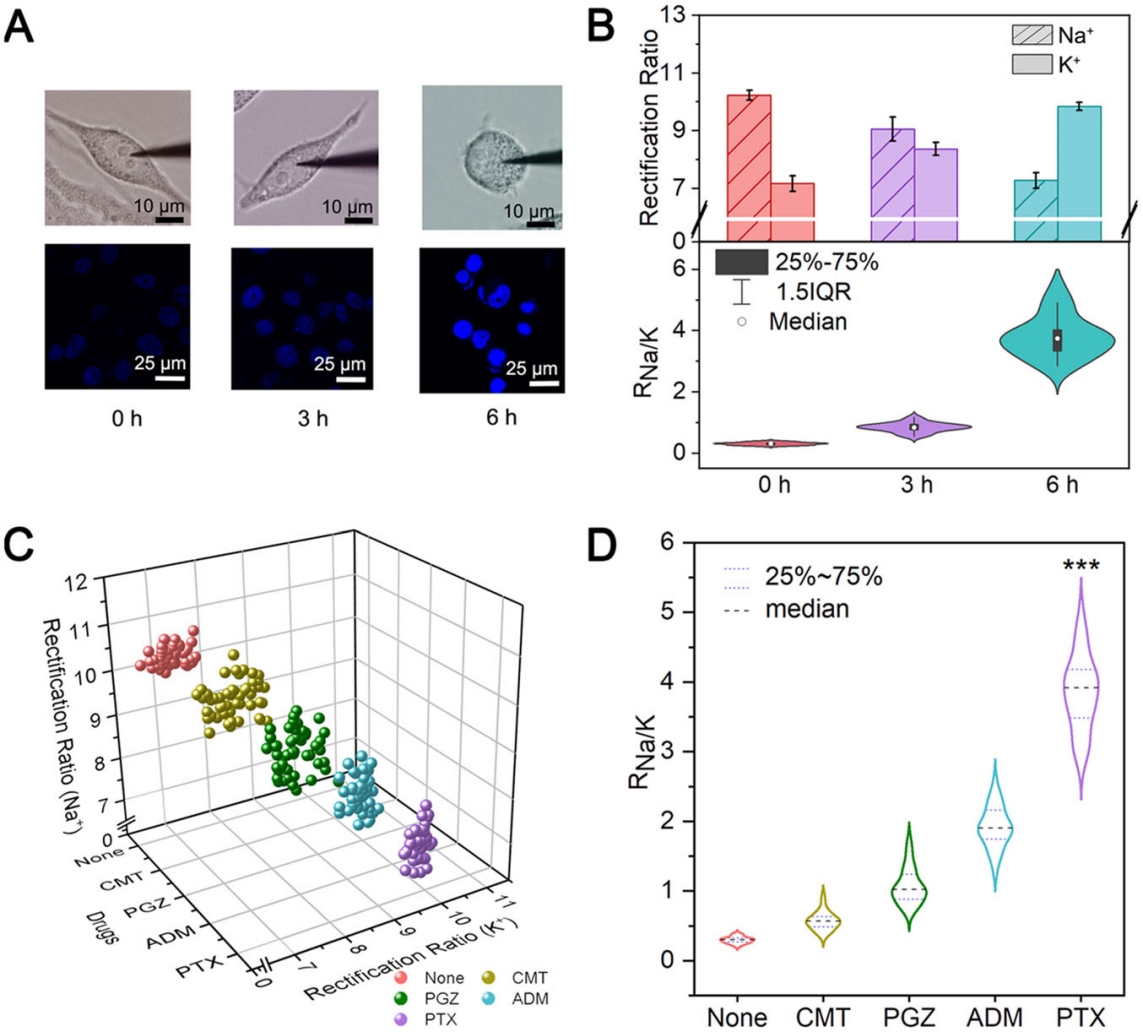
(A) The bright‐field micrographs and corresponding confocal fluorescence micrographs of DAPI‐stained PC‐3 cells. (B) ICR ratios (upper) of respective Na‐nanopores and K‐nanopores and the corresponding R_Na/K_ (below) of 20 PC‐3 cells after treating by 50 µM PTX for 0, 3, and 6 h. (C) The ICR ratios of respective Na‐nanopores and K‐nanopores and (D) the corresponding R_Na/K_ of 50 PC‐3 cells upon stimulation with CMT, PGZ, ADM and PTX for 6 h. *P ≤ 0.05, **P ≤ 0.01, ***P ≤ 0.001 by Student's *t‐*test

The nanotool was then implemented for R_Na/K_ detection in a single living cell. Although the non‐destructiveness of single barreled nanopipette has been revealed by previous dye staining studies, the good biocompatibility of present θ‐nanopipette was also identified (Figures S8–S10). The dynamic change of intracellular R_Na/K_, corresponding to the gradient reversal of cellular Na^+^ and K^+^, would take place in PSAVD, accompanied by the decrease in the cell volume and nuclear condensation.[^4b,4d^ ^]^ Assisted by 4,6‐diamidino‐2‐phenylindole (DAPI) staining,^[^ ^20^ ^]^ the drug of paclitaxel (PTX) was then used to stimulate the PSAVD of PC‐3 cell, and the R_Na/K_ of which was probed by the as‐developed nanotool. As shown in Figure 3A, 3 h PTX treatment could not cause noticeable volume change of the PC‐3 cell but slight fluorescence enhancement, while 6 h treatment induced distinct volume loss and the chromatin condensation as reflected by DAPI dye, indicating the occurrence of PSAVD (Figures S11, S12). More clearly, as revealed by the nanotool shown in Figure 3B upper, the ICR ratios of the Na‐nanopores exhibited obvious decrease from ∼10.23 to ∼9.05 upon 3 h treatment, which further significantly declined to ∼7.27 upon 6 h treatment. By contrast, the ICR ratios of the K‐nanopores exhibited a quite opposite trend from ∼7.16 to ∼8.36, and then to ∼9.84. The statistical analysis of 20 PC‐3 cells per group was then conducted. As shown in Figure 3B below, the R_Na/K_ exhibited stepwise increase from ∼0.31 to ∼0.84 and then to ∼3.80, accompanied by a wider distribution with interquartile range (IQR) of ∼0.10, 0.22, and 0.73, respectively. The feasibility of the nanotool for differentiating drug effects was then performed by targeting 50 PC‐3 cells per group using PTX, camptothecin (CMT), pioglitazone (PGZ), and adriamycin (ADM). As shown in Figure 3C, variable drug effects as reflected by ICR ratios of Na‐nanopores and K‐nanopores could be observed as compared to the non‐treated cells. Especially, as derived in Figure 3D, the R_Na/K_ corresponding to CMT, PGZ, ADM, PTX were 0.57 ± 0.13, 1.08 ± 0.31, 1.96 ± 0.36, and 3.90 ± 0.63, respectively, with the enhanced significant difference. Besides, the R_Na/K_ during ultraviolet‐induced PSAVD was also investigated (Figure S13). These results demonstrated the potential of our nanotool for R_Na/K_‐associated drug evaluation.

### 2.3 Intracellular R_Na/K_ associates with metastatic potential

**FIGURE 4:**
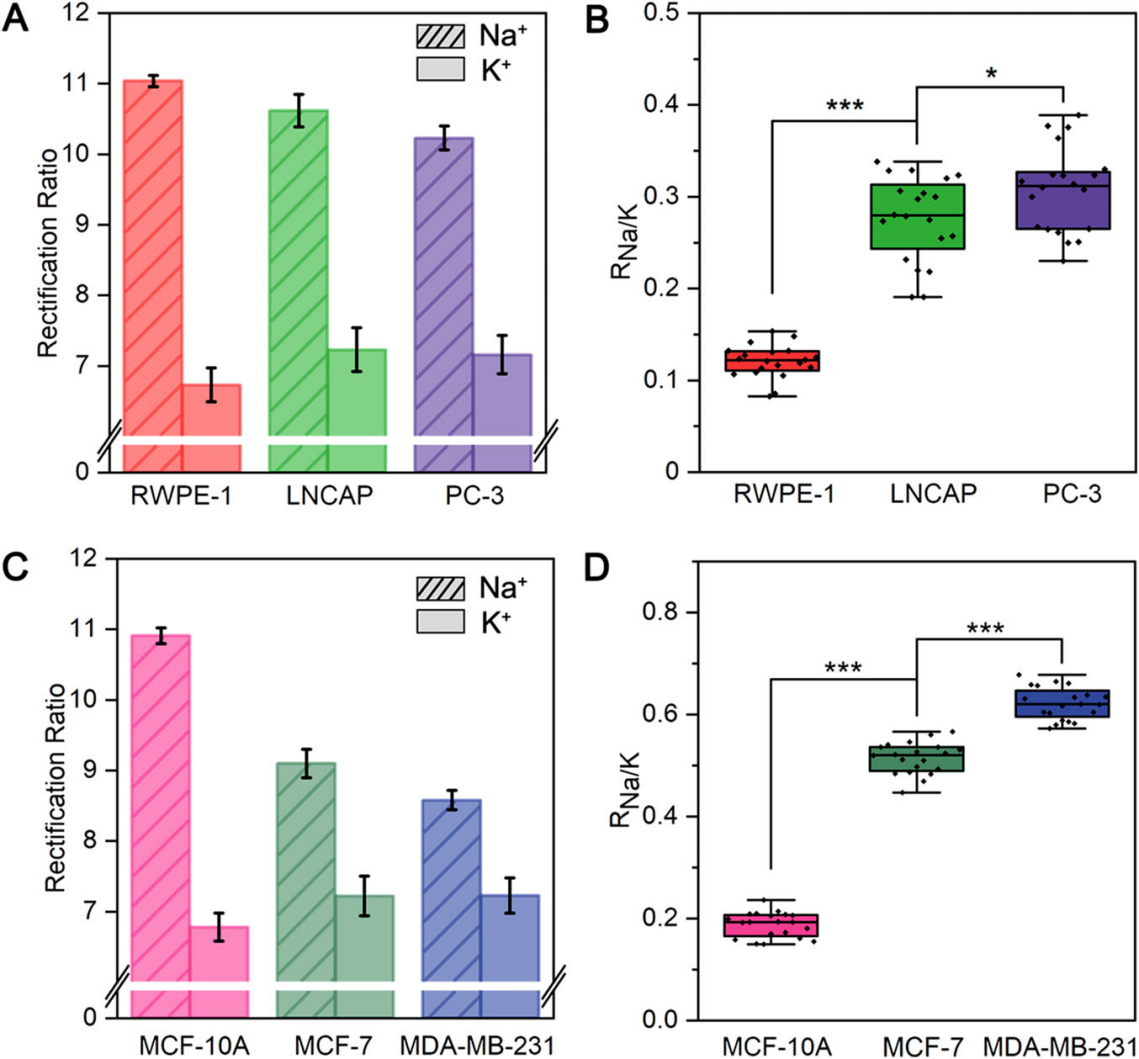
(A) The ICR ratios of Na‐nanopores and K‐nanopores and (B) the corresponding R_Na/K_ toward three cell lines of human prostate cells. (C) The ICR ratios of Na‐nanopores and K‐nanopores and (D) the corresponding R_Na/K_ toward three cell lines of human breast cells. Error bars represent standard deviations from twenty independent tests. *P ≤ 0.05, **P ≤ 0.01, ***P ≤ 0.001 by Student's *t*‐test

The intracellular R_Na/K_ in two kinds of cells, that is, human prostate cells and human breast cells with different metastatic potential, were probed for investigating the role of R_Na/K_ in human cancer metastasis. Three human prostate cells, that is, non‐metastatic RWPE‐1, metastatic LNCAP, and highly metastatic PC‐3 cells were addressed. As shown in Figure 4A, the ICR ratios of Na‐nanopores and K‐nanopores exhibited an observable difference among the three cell lines. The ICR ratios of the Na‐nanopore in PC‐3 were lower than those of LNCAP and RWPE‐1, corresponding to 10.23, 10.62, and 11.04, respectively, with the corresponding ICR ratios of K‐nanopores as 7.16, 7.23, and 6.73. As converted in Figure 4B, the corresponding R_Na/K_ were 0.12 ± 0.02, 0.28 ± 0.05, and 0.31 ± 0.05, which showed a tendency of increasing with the increased metastatic potential of cells. We speculate that such a phenomenon was due to a net increase of the intracellular Na^+^ resulting from the inhibition of Na^+^/K^+^‐ATPase pump in cancerous cells.^[4c^ ^]^ To confirm that, another three cell lines of human breast cells with different metastatic potential, that is, non‐metastatic MCF‐10A, metastatic MCF‐7, and highly metastatic MDA‐MB‐231 cells, were investigated. As shown in Figure 4C,D, similar trend was also observed, confirming the correlation of metastatic potential with the cellular R_Na/K_. These results unveiled the potential of this θ‐nanopipette tool for understanding the role of R_Na/K_ in various physiological and pathological processes.

## 3 CONCLUSION

To conclude, we have devised a single‐cell R_Na/K_ detector by integrating functional nucleic acids and θ‐nanopipette. The respective functionalization and simultaneous implementation of the two independent nanopores create a simple approach of detecting the cytosolic R_Na/K_ in a single living cell. Practical intracellular studies confirmed the applicability and minimal invasiveness of this θ‐nanopipette nanotool in single‐cell R_Na/K_ studies with cell‐context preservation as well as its potential in R_Na/K_‐associated drug evaluation. Besides, R_Na/K_ had been shown to be closely correlated with the metastatic potential of cancerous cells. This work has not only provided an accessible single‐cell R_Na/K_ sensor, but also enriched current palette of θ‐nanopipette nanotools and is envisioned to inspire more interest in exploration of novel application scenarios of θ‐nanopipettes.

## 4 EXPERIMENTAL SECTION

### 4.1 Reagents and materials

θ‐type quartz capillaries with filament (O.D.: 1.2 mm, I.D.: 0.9 mm; 7.5 cm length) were provided from Sutter Instrument. Dimethyl sulfoxide, tris(2‐carboxyethyl)‐phosphine hydrochloride (TCEP), and 6‐hydroxy‐1‐hexanethiol (MCH) were purchased from Sigma‐Aldrich (U.S.A). PGZ was supplied from Tianjing Xien Si Opod Technology Co., Ltd. CMT was purchased from Shanghai Yuanye Bio‐Technology Co., Ltd. PTX was purchased from Jiangsu Aikon Biopharmaceutical R&D Co., Ltd. MDA‐MB‐231 cells, MCF‐7 cells, MCF‐10A cells, LNCAP cells, RPMI‐1640 medium, F‐12K medium, Leibovitz's L‐15 medium, 0.25% trypsin/2.2 mM EDTA solution, Hoechst 33342 (H342), propidium iodide (PI), DAPI, ADM, and ethidium bromide green dye (EB) were provided from KeyGen Biotech. Co. Ltd. (Nanjing, China). RWPE‐1 cells, PC‐3 cells, mammary epithelium basal medium (MEBM), RWPE‐1 specialized medium, hydrocortisone (HCT), human epidermal growth factor (hEGF), insulin, cholera toxin (CT), bovine pituitary extract (BPE) and gibberellic acid (GA) were purchased from iCell Bioscience, Inc. (Shanghai, China). Fetal bovine serum (FBS) was supplied from Glibco (USA). All other chemicals, of analytical grade, were offered from Sigma Aldrich and used as received. All aqueous solutions were prepared from ultrapure water (Millipore, 18 MΩ cm^–1^). 15%, 4% ‐ 15% precast‐glgel hepes native‐PAGES and the DNA sequences (Table 1) were provided from Sangon Biotech. Co., Ltd. (Shanghai, China).

**TABLE 1:** The sequences of DNA employed in this work

Name	Sequences (5′‐3′)
Thiol‐DNA (TDNA)	5′‐SH‐(CH_2_)_6_‐TCATCGAGAGAGAGATCCTC‐3′
K^+^‐aptamer (ADNA)	5′‐CTCTCTGGTGGTGGGGGGGGAAGGATGGGTGTCTTCTCTCTC‐3′
NaA43S	5′‐SH‐(CH_2_)_6_‐ACAGTGCGTGAACTCTATCTATrAGGAAGTACCGCCGC‐3′
NaA43E	5′‐GCGGCGGTACCAGGTCAAAGGTGGGTGAGGGGACGCCAAGAGTCCCCGCGGTTAGATAGAG‐3′

### 4.2 Experimental setup and data acquisition

The electrochemical recordings were carried out by a CHI 760C electrochemical workstation (CH Instrument, Shanghai, China). The monitoring of the current–voltage (*I*–*V*) curves was performed by using the scan rate of 50 mVs^–1^ and the voltage sweeping from −1.0 to 1.0 V. The θ‐nanopipettes employed in the work were fabricated by a P‐2000 laser‐pulling (Sutter Instruments, USA). The θ‐nanopipette is anchored to the holder (Axon Instruments, USA), and further precisely controlled by a MP‐225 micromanipulator (Sutter Instrument, USA) under the observation of Olympus inverted fluorescence microscope (Ti2‐E, Nikon, Japan). Before electrochemical measurements, the tip of the θ‐nanopipette was backfilled by 20 mM Tris‐HCl (pH 7.4) with LiCl of 40 mM and checked under the microscope for the exclusion of the air bubbles. Field‐emission scanning electron microscopic (FE‐SEM) images were collected by a S‐4800 Instrument (Hitachi Co., Japan). Au coating was carried out with the vacuum evaporation equipment (ACE600, Leica, Germany). The liquid‐filled θ‐nanopipettes were vacuumed in the small transition chamber next to the glove box (Vigor, China). Confocal laser scanning microscopy (CLSM) characterization was performed using a Leica TCS SP5 microscope (Germany).

### 4.3 Fabrication of Au deposited θ‐nanopores

The θ‐type quartz capillaries (QT 120‐90‐7.5), consisting of an inner diameter of 0.90 mm and an outer diameter of 1.2 mm, were firstly cleaned with plasma for removing the dust and activating the silicon hydroxyl group of the capillaries. The θ‐nanopores were then pulled by the P‐2000 laser‐pulling using the pulling parameters. The first line of the procedure: Heat = 860, FIL = 4, Vel = 30, Del = 190, Pul = 40; The second line of the procedure: Heat = 900, FIL = 3, Vel = 20, Del = 140, Pul = 150. To guarantee the reproducibility of the aperture geometry, the pulling time between two θ‐nanopores should be varied within 0.1 s. The SEM instrument was used for aperture characterization. Afterward, the tip of the θ‐nanopores was coated by gold film with 10 nm thick from the angle of 30° sputtering.

### 4.4 Synthesis of Na‐probe and K‐probe

The Na‐probe was synthesized by subsequently mixing the substrate and enzyme strands (1:1.1 ratio), heated to 80°C for 3 min, and then cooled to ambient temperature for more than 30 min to guarantee the hybridization between NaA43S and NaA43E.^[^ ^14^ ^]^ The K‐probe was synthesized by mixing the Thiol‐DNA (TDNA) of 20 µM and K^+^‐aptamer (ADNA) of 20 µM in buffer solution (pH 7.4, 20 mM Tris‐HCl) and incubating for 60 min.^[15b^ ^]^ The obtained Na‐probe and K‐probe were further characterized by gel electrophoresis.

### 4.5 Functionalization of θ‐nanopipette

The Na‐probe was firstly mixed with TCEP and incubated for 30 min. Then 10 µM activated Na‐probe was added into one nanopore by an Eppendorf Microloader for 16 h reaction with Au layer on the interior surface.^[9b^ ^]^ The same modification step was employed for establishing the K‐nanopore. Afterward, the θ‐nanopipette was backfilled and immersed with 1 mM MCH for 10 min. Note that the θ‐nanopipette was rinsed repeatedly with the buffer solution between the modification steps.

### 4.6 Cell culture and induction of apoptosis

MCF‐10A cells were grown in the MEBM medium containing BPE (0.4% v/v in MEBM), hEGF (0.1% v/v in MEBM), Insulin (0.1% v/v in MEBM), HCT (0.1% v/v in MEBM), GA (0.1% v/v in MEBM) and CT (0.1% v/v in MEBM) and maintained at 37°C in a humid atmosphere containing 5% CO_2_/ 95% air within 24 h culture. PC‐3 cells were cultured in F‐12K Medium in the presence of 10% FBS, penicillin (80 U/ml) and streptomycin (0.08 mg/ml). Afterward, the PC‐3 cells were maintained at the same atmosphere as above. LNCAP and MCF‐7 cells were grown in the 1640 medium containing streptomycin (0.08 mg/ml), penicillin (80 U/ml), and 10% FBS at 37°C in a humid atmosphere (5% CO_2_/ 95% air). RWPE‐1 cells were growth in the specialized medium containing 0.2% growth factor and 1% antibiotics. MDA‐MB‐231 cells were growth in L‐15 medium containing 10% FBS. The experiments of cell penetration and CLSM were performed after the adherence of the cells to the culture dish. Afterward, the adherent PC‐3 cells were stimulated by drugs (50 µM PTX, 50 µM CMT, 50 µM PGZ and 50 µM ADM) and ultraviolet light with different time for the following experiment.

### 4.7 Single‐cell experiments

The ionic current measurements were processed using the CHI 760C electrochemical equipment. First of all, the θ‐nanopipette tool is installed to the holder and further operated with the inverted fluorescence microscope and a three‐dimensional MP‐225 manipulator. Before the experiments, cells were rinsed three times and maintained under the similar osmotic pressure environment of the cells (pH 7.4, 20 mM Tris‐HCl, 40 mM LiCl). The θ‐nanopipette tool was precisely and stably operated by the MP‐225 manipulator with a submicron resolution of 62.5 nm and observed under the assistance of inverted fluorescence microscope for successively immersing the solution and inserting into the cell. The θ‐nanopipette tool was controlled with coarse movement mode before entering the solution, and then changed to fine movement mode until arriving onto the cell membrane with the deformation of the cell, the position of which was marked as 0. Afterward, the nanotool was lowered 0.5 µm at *Z*–axis by ultra‐fine mode and maintained in the single cell for 10 s, then it was withdrawn fastly from the cell and subsequently placed in another detection solution (pH 7.4, 20 mM Tris‐HCl, 40 mM LiCl) for electrochemical measurements.

### 4.8 Cell vitality measurement

Firstly, the cell morphology was monitored per 20 min for 100 min under the inverted fluorescence microscope after insertions five times by the θ‐nanopipette tool, and the corresponding images were taken continuously by the camera software on the computer. After long‐time insertion of the θ‐nanopipette tool, the membrane integrity and viability of the cell were respectively verified by PI and H342 dyes. The PC‐3 cells were respectively stained using these two dyes according to the instructions. The fluorescence images of the cells were captured before and after penetration and withdrawal, the cells should be seeded into the small dishes and incubated for 12 h before initiation of cell experiments.

### 4.9 Confocal microscopy

The 2×10^5^ cells/ml PC‐3 cells were grown in 35 cm glass‐bottom confocal dishes (Cellvis) and incubated for 24 h. After the PTX stimulation for different times, the cells were rinsed with PBS (pH 7.4) and then stained by 10 µg/ml DAPI for 5 min incubation at dark atmosphere. Before confocal imaging, the cells were rinsed with PBS and then observed under oil immersion lens (100×) by the excitation wavelength of 380 nm. The gain setting was kept constant for all the samples.

### 4.10 Statistical analysis

All data were representative results from biologically independent experiments. The experimental results were exhibited as mean ± SD. Statistical analysis was performed by the Student's *t*‐test (*P ≤ 0.05, **P ≤ 0.01, ***P ≤ 0.001).

## CONFLICT OF INTEREST

The authors declare no conflict of interest.

## Supporting information



Supporting Information

## Data Availability

The data that supports the findings of this study are available in the Supporting Information of this article.
