# Supplementary material for: Functional nucleic acid engineered double‐barreled nanopores for measuring sodium to potassium ratio at single‐cell level
Source: Exploration (Beijing). 2022 May 23;2(5):20220025. doi: 10.1002/EXP.20220025 (PMC10190848; doi:10.1002/EXP.20220025)
Supplement: Supplementary file 1 — Supporting Information [file EXP2-2-20220025-s001.docx]

Supporting Information

Functional Nucleic Acid Engineered Double Barreled Nanopores for Measuring Sodium to Potassium Ratio at Single-Cell Level

Xiao-Mei Shi, Fang-Qing Liu, Bing Wang, Si-Yuan Yu, Yi-Tong Xu, Wei-Wei Zhao,* Dechen Jiang, Hong-Yuan Chen, and Jing-Juan Xu*

Dr. Xiao-Mei Shi, Fang-Qing Liu, Bing Wang, Si-Yuan Yu, Yi-Tong Xu, Prof. Wei-Wei Zhao, Prof. Dechen Jiang, Prof. Hong-Yuan Chen, Prof. Jing-Juan Xu

State Key Laboratory of Analytical Chemistry for Life Science, School of Chemistry and Chemical Engineering, Nanjing University, Nanjing 210023. P. R. China

E-mail: zww@nju.edu.cn; xujj@nju.edu.cn

Contents

Results and discussion

Figure S1. The schematic diagrams of (A) depositing Au layer by the magnetron sputtering and (B) the θ-nanopipette before and after Au coating.

Figure S2: Gel electrophoresis confirming the formation of (A) Na-probe and (B) K-probe.

Figure S3: OCP measurements of (A) left Na-nanopore and (B) right K-nanopore.

Figure S4: The control experiments performed without (A) Na-probe and (B) K-probe modification.

Figure S5: The stability of the nanotool.

Figure S6: Optimization of incubation time for (A) 200 mM Na^+^ and (B) 200 mM K^+^ reaction.

Figure S7: The parallel responses of 20 nanotools fabricated in the same batch.

Figure S8: Continuous bright-field images taken per 20 min within 100 min.

Figure S9: Bright-field and fluorescence images of PI-stained PC-3 cells.

Figure S10: Bright-field and fluorescence images of H342-stained PC-3 cells.

Figure S11: Confocal laser scanning microscopy of PC-3 cells after PTX treatment.

Figure S12: Confocal laser scanning microscopy of PC-3 cells after 0.5 % DMSO treatment.

Figure S13: The effect of ultraviolet light on PC-3 cells.

References

Results and discussion

As shown in **Figure S1A** and **Figure S1B**, the θ-nanopipettes were obtained by laser-pulling of θ-type capillaries and the interior walls were then decorated with Au layer by magnetron sputtering from the tip side with an angle of 30° to the Au source.


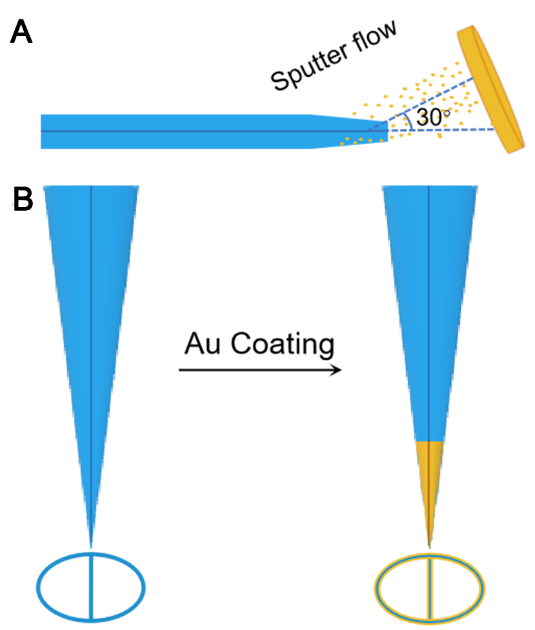


**Figure S1.** The schematic diagrams of (A) depositing Au layer by the magnetron sputtering and (B) the θ-nanopipette before and after Au coating.

As shown in **Figure S2A**, lanes 1, 2 and 3 are observed for 20 bp marker, NaA43S and NaA43E, respectively. A new band (lane 4) with larger molecular weight (MW) would appear, indicating the formation of Na-probe. The similar phenomenon was shown in **Figure S2B**, compared to the Thiol-DNA (TDNA) sequences (lane 2) and the K^+^-aptamer (ADNA) sequences (lane 3), the K-probe (lane 4) exhibited the higher MW.


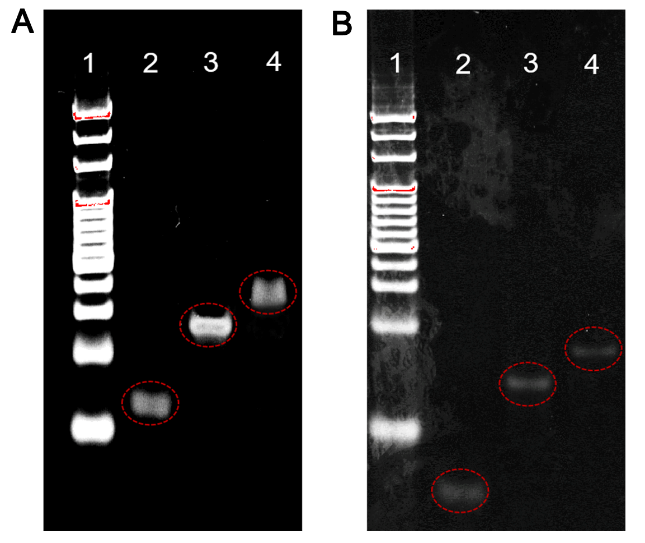


**Figure S2.** Gel electrophoresis confirming the formation of (A) Na-probe and (B) K-probe. The gel was stained with ethidium bromide (EB) Green Dye for visualization.

As shown in **Figure S3**, the dramatic open-circuit potential (OCP) change confirmed the successful functionalization of the DNA sequences within the Au-deposited θ-nanopipette.


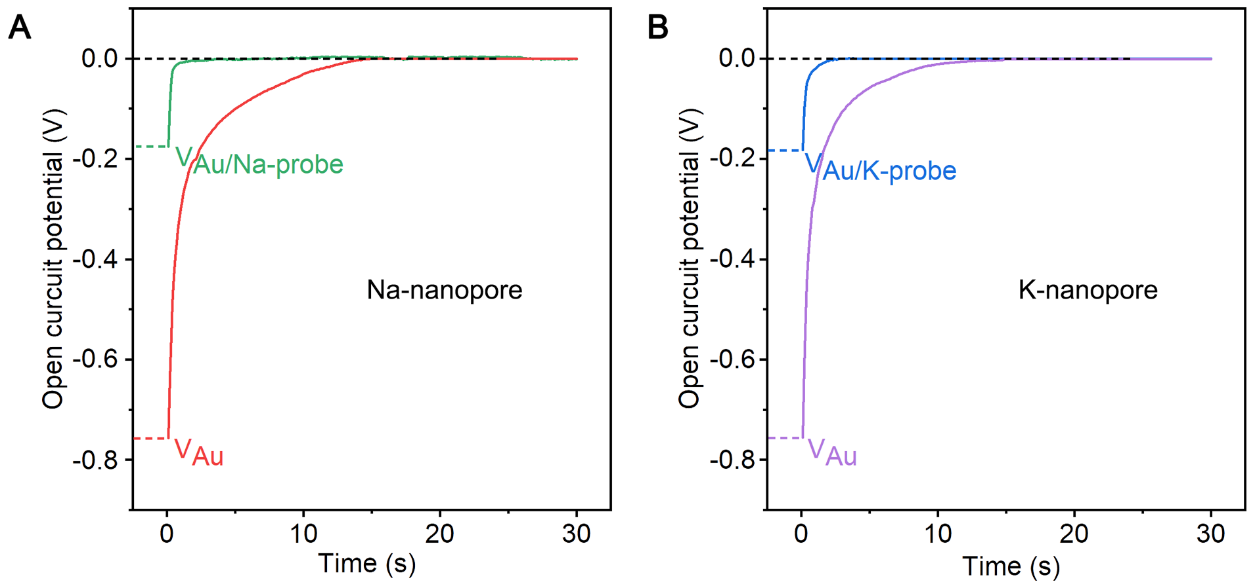


**Figure S3.** OCP measurements of (A) left Na-nanopore and (B) right K-nanopore.

The control experiments were performed on the prepared θ-nanopipette without the modification of Na-probe and K-probe. As shown in **Figure S4A**, the ICR signals exhibited negligible difference before and after the incubation with 100 mM Na^+^. The similar results was obtained for incubating with 100 mM K^+^ (**Figure S4B**).


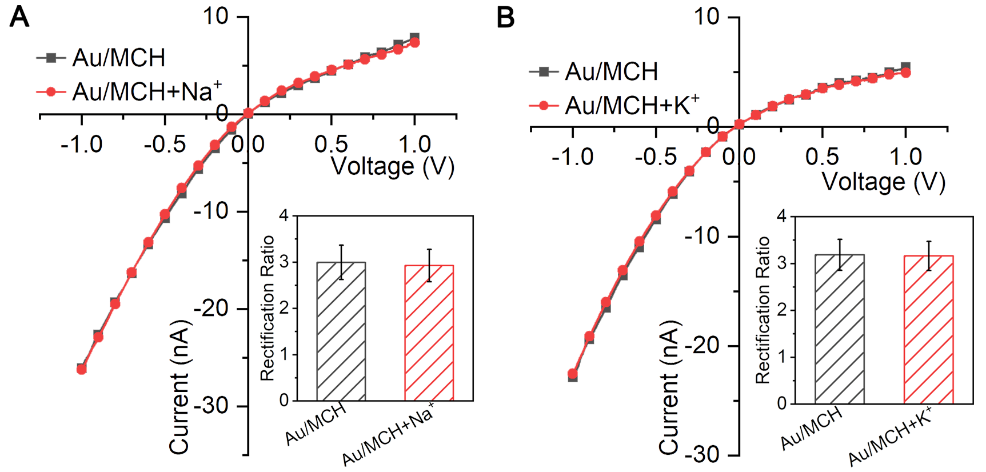


**Figure S4.** The control experiments performed without (A) Na-probe and (B) K-probe modification. Insets show the corresponding ICR ratios.

Furthermore, the stability of nanotool was studied. Specifically, the Na-nanopore (**Figure S5A**) and K-nanopore (**Figure S5B**) were respectively studied in electrolyte for different time: 0 h, 12 h, 24 h, 36 h and 48 h. The nearly unchanged ICR signals indicated the stability of the as-fabricated nanotool.


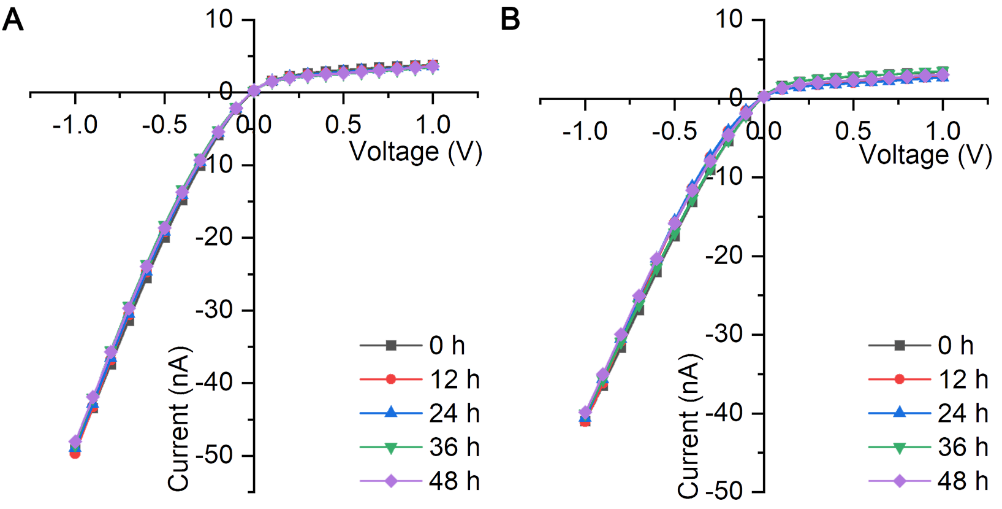


**Figure S5.** The stability of the nanotool.

As shown in **Figure S6**, the exposing time of the nanotool was optimized in the presence of 200 mM Na^+^ and 200 mM K^+^, respectively. The ICR signal descended rapidly with the increase of the exposing time up to 10 s and then tended to slow down and be constant. The optimized time of 10 s was then chosen for the following experiments.


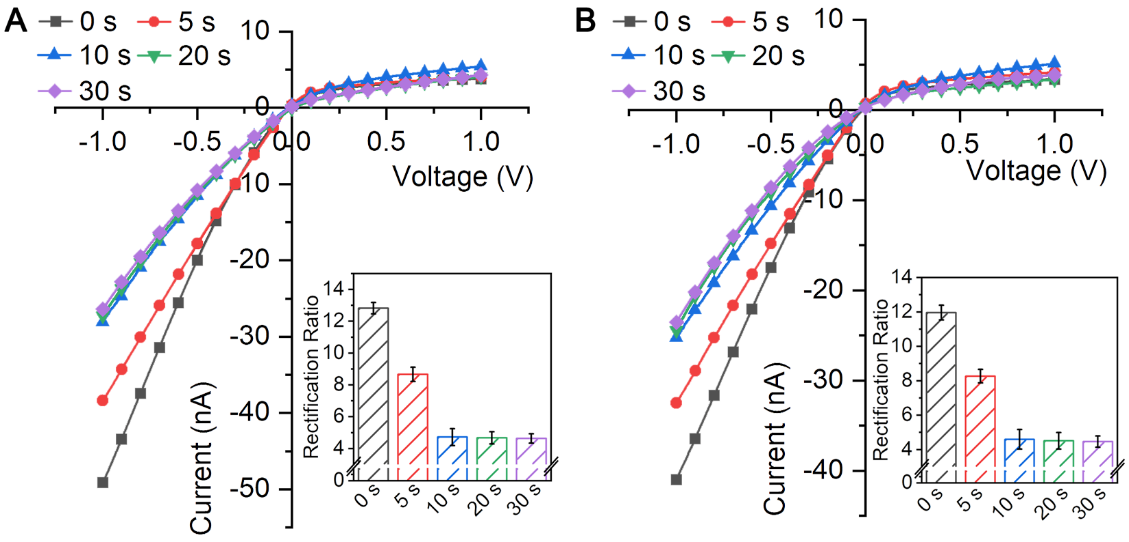


**Figure S6.** Optimization of incubation time for (A) 200 mM Na^+^ and (B) 200 mM K^+^ reaction. Insets show the corresponding ICR ratios.

As shown in **Figure S7A** and **Figure S7B**, parallel responses of the nanotools prepared under same conditions were then investigated. The Na-nanopore with -49.1 ± 0.3 nA (at -1.0 V), 3.8 ± 0.1 nA (+1.0 V) and the K-nanopore with -40.9 ± 0.3 nA (at –1.0 V) and 3.4 ± 0.1 nA (+1.0 V) were kept, others were discarded and not used for the intracellular application.


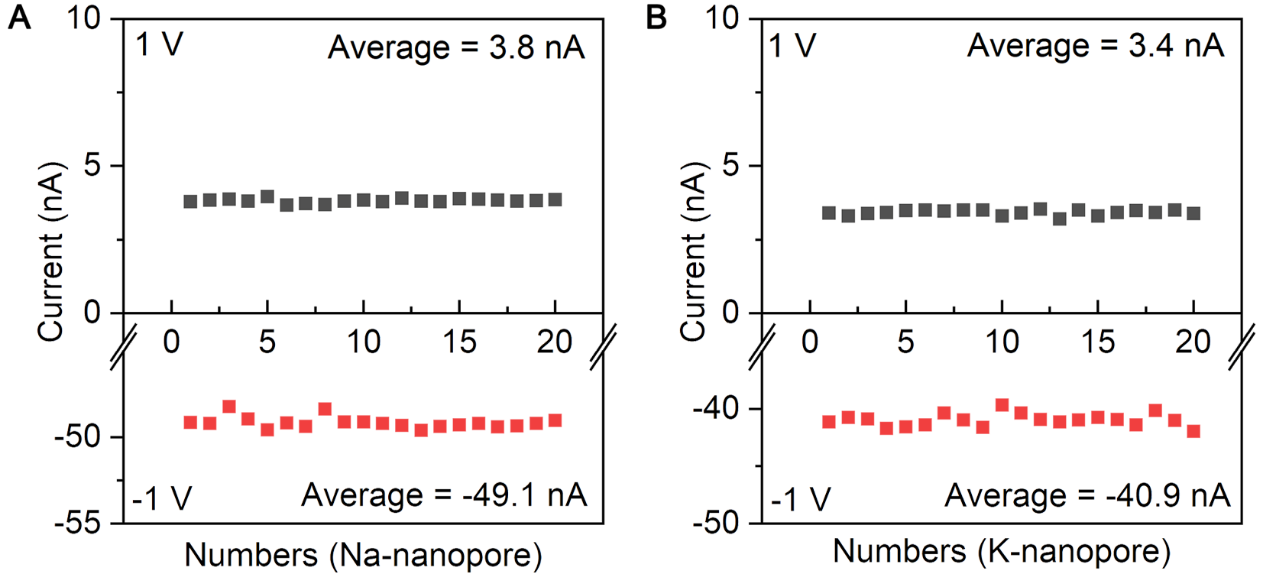


**Figure S7.** The parallel responses of 20 nanotools fabricated in the same batch.

The cell morphology was continuously observed for 100 min under microscope after cell insertions for five times. As shown in **Figure S8**, the morphology of the targeted cell maintained well, indicating no interruption against cell morphology by the nanotool.


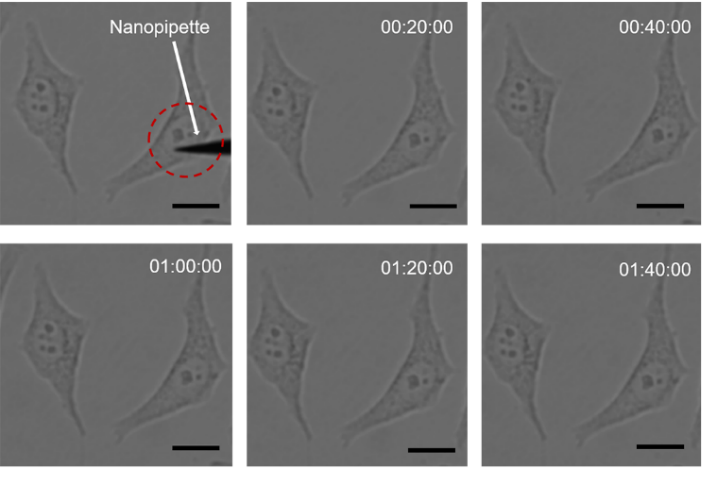


**Figure S8.** Continuous bright-field images taken per 20 min for 100 min to show the cell morphology after repeated insertions into the cell for five times. Scale bar: 20 µm.

The membrane integrity of the penetrated cell was also verified after long-time insertion and withdrawal by utilizing the fluorescent dye propidium iodide (PI).^[1]^ This dye has no membrane impermeability with living cells, but can stain the dead cells and exhibit strong red fluorescence. The fluorescence micrographs of PI-stained PC-3 cells before and after penetration and withdrawal of the nanotool were shown in **Figure S9**. No red fluorescence was shown before and after penetration and withdrawal, indicating the good membrane integrity of the cell.


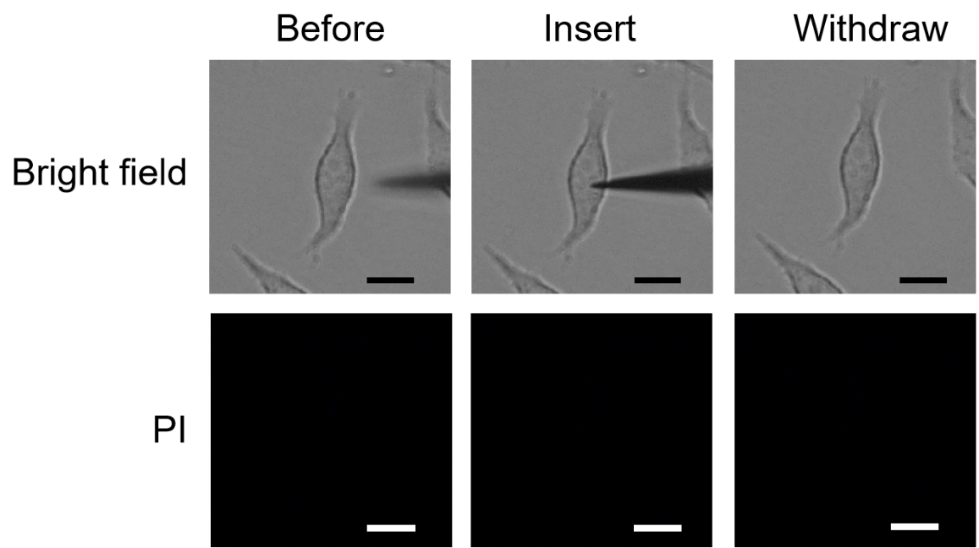


**Figure S9.** Bright-field and fluorescence images of PI-stained PC-3 cells before and after the penetration and withdrawal of the nanotool. The excitation wavelength was 536 nm. Scale bar: 20 µm.

In addition, Hoechst 33342 (H342) was used to verify the cell viability. The H342 dye^[2]^ possessed a certain membrane permeability. The normal cell could be slightly stained and exhibit low blue fluorescence, while the dead cells could exhibit bright blue fluorescence. As shown in **Figure S10**, the fluorescence images of the cells were captured per 10 min for 60 min and remained unchanged before and after penetration and withdrawal, indicating the viability maintenance of cells by long-time penetration and withdrawal of nanotool.


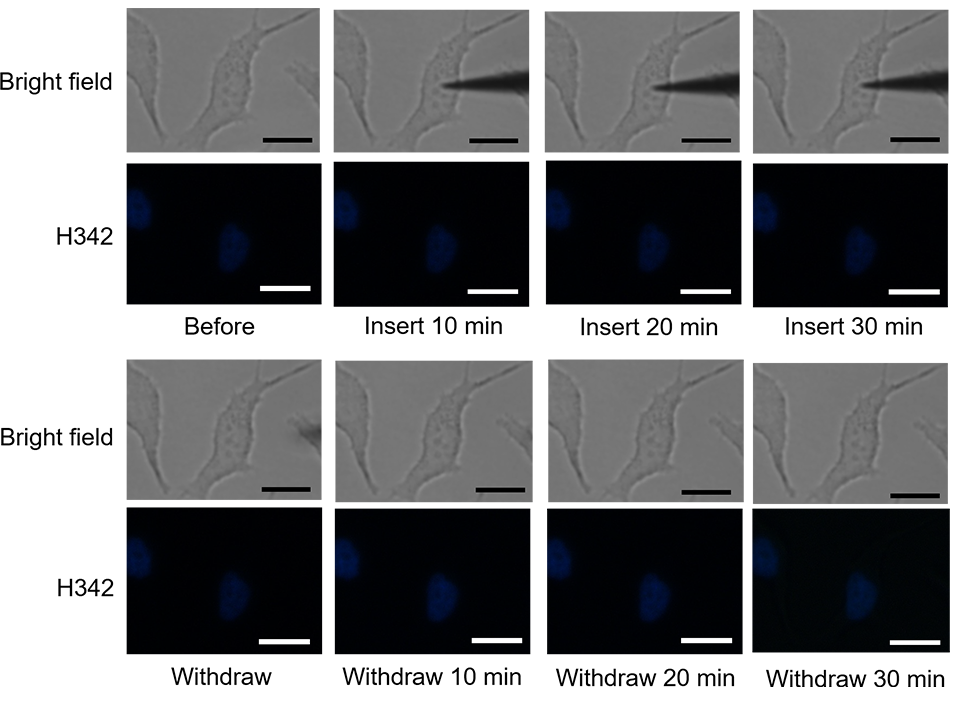


**Figure S10.** Bright-field and fluorescence images of the H342-stained PC-3 cells captured per 10 min for 60 min before and after penetration and withdrawal of the nanotool. The excitation wavelength was 352 nm. Scale bar: 20 µm.

As shown in **Figure S11**, 6 h treatment by 50 µM paclitaxel (PTX) resulted in the enhanced loss of the cellular volume and the chromatin condensation as reflected by the 4’,6-diamidino-2-phenylindole (DAPI) fluorescence,^[3]^ suggesting the occurrence of apoptotic volume decrease (PSAVD) of the PC-3 cells.


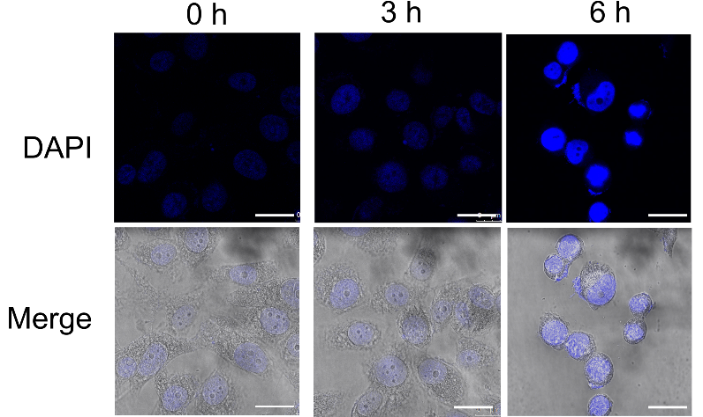


**Figure S11.** Confocal laser scanning microscopy of PC-3 cells after PTX treatment for 0 h, 3 h, 6 h under oil immersion lens (100x). The excitation wavelength was 380 nm. Scale bar: 25 µm.

The control experiment was carried out after the treatment by 0.5 % dimethyl sulfoxide (DMSO) solvent for the same time intervals. As shown in **Figure S12**, the cells exhibited low blue fluorescence and the nuclei was almost round, indicating no influence against the cells by 0.5 % DMSO solvent.


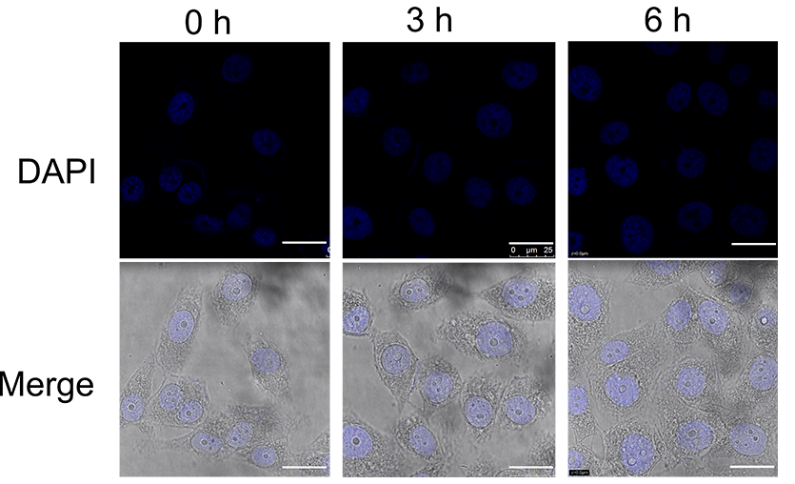


**Figure S12.** Confocal laser scanning microscopy of PC-3 cells after 0.5 % DMSO treatment for 0 h, 3 h, 6 h under oil immersion lens (100x). The excitation wavelength was 380 nm. Scale bars: 25 µm.

The ultraviolet light was also used to stimulate the primary stage of PSAVD of PC-3 cell, and the R_Na/K_ of which was probed by the as-developed nanotool. As shown in **Figure S13A**, 2 h ultraviolet could not cause noticable volume change of the PC-3 cell, while 4 h treatment induced distinct volume loss, indicating the occurrence of the PSAVD. More clearly, as revealed by the nanotool shown in **Figure S13B**, the ICR ratios of the Na-nanopore exhibited obvious decrease from ca. 10.25 to ca. 9.26 upon 2 h treatment, which further significantly declined to ca. 7.25 upon 4 h treatment. By contrast, the ICR ratios of the K-nanopores exhibited a quite opposite trend from ca. 7.18, to ca. 8.20, and then to ca. 9.82. As shown in **Figure S13C**, the R_Na/K_ exhibited stepwise increase from ca. 0.30 to ca. 0.73 and then to ca. 3.75, accompanied by a wider distribution with interquartile range (IQR) of ca. 0.09, 0.24 and 0.93, respectively.


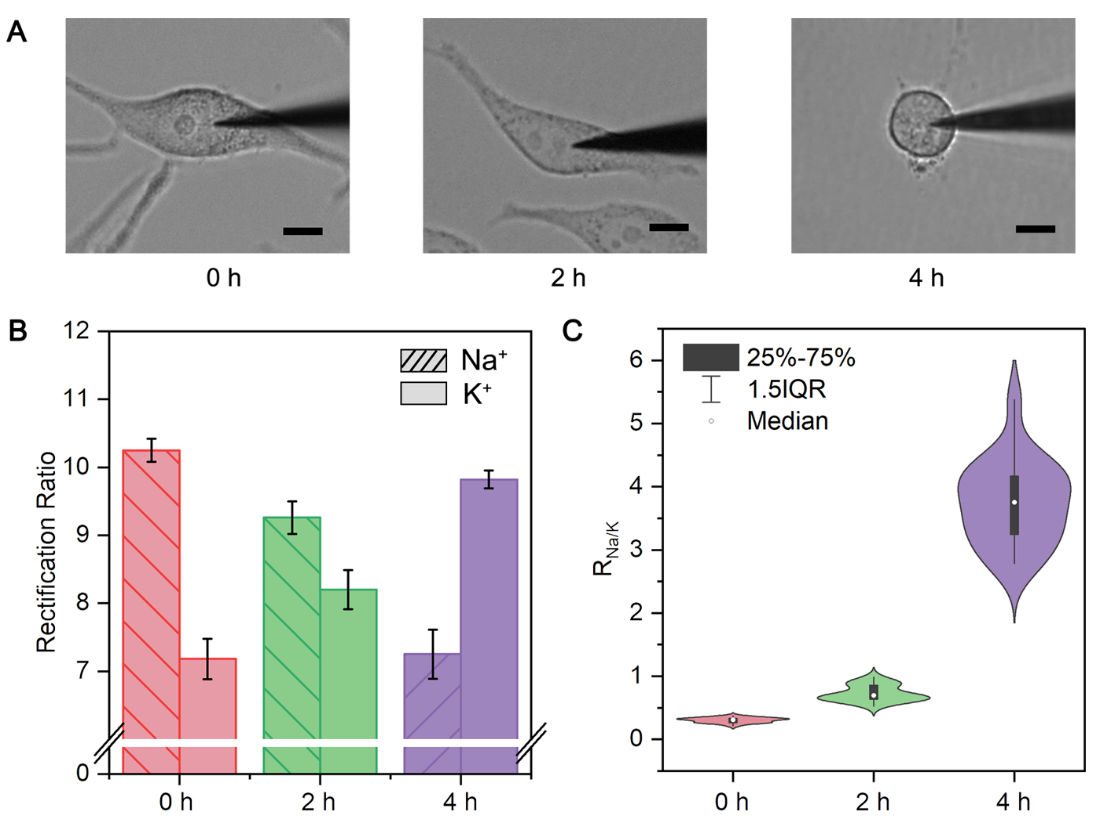


**Figure S13.** The effect of ultraviolet light on PC-3 cells. (A) Bright-field images of single PC-3 cell, (B) ICR ratios of respective Na-nanopores and K-nanopores and corresponding (C) R_Na/K_ of 20 PC-3 after treatment by ultraviolet light for 0 h, 2 h and 4 h. Scale bar: 10 µm.

**3. References**

[1] L. Z. Jiang, R. Tixeira, S. Caruso, G. K. Atkin-Smith, A. A. Baxter, S. Paone, M. D. Hulett, I. K. Poon, *Nat. Protoc.* **2016**, *11*, 655.

[2] M. G. Tian, J. Sun, B. L. Dong, W. Y. Lin, *Angew. Chem. Int. Ed*. **2018**, *57*, 16506.

[3] a) S. T. Wang, H. Wang, J. Jiao, K. J. Chen, G. E. Owens, K. I. Kamei, J. Sun, D. J. Sherman, C. P. Behrenbruch, H. Wu, H. R. Tseng, *Angew. Chem. Int. Ed*. **2009**, *48*, 8970; b) I. Yeh, A. von Deimling, B. C. Bastian, *J. Natl. Cancer Inst.* **2013**, *105*, 917.
